# Single-cell Glycogenomics Deciphers Links Between Altered Transcriptional Regulation and Aberrant Glycosylation in Alzheimer’s Disease

**DOI:** 10.1101/2023.12.25.573290

**Published:** 2023-12-27

**Authors:** Yusuke Matsui, Akira Togayachi, Kazuma Sakamoto, Kiyohiko Angata, Kenji Kadomatsu, Shoko Nishihara

**Affiliations:** 1 Institute for Glyco-core Research (iGCORE), Nagoya University, Furo-cho, Chikusa-ku, Nagoya 464-8601, Japan; 2 Biomedical and Health Informatics Unit, Department of Integrated Health Science, Nagoya University Graduate School of Medicine, Daiko-minami, Higashi-ku, Nagoya, 461-8673, Japan; 3 Glycan and Life Systems Integration Center (GaLSIC), Soka University, 1-236 Tangi-machi, Hachioji, Tokyo 192-8577, Japan; 4 Department of Biochemistry, Nagoya University Graduate School of Medicine, Tsurumai-cho, Showa-ku, Nagoya, 466-8550, Japan

## Abstract

Glycosylation is increasingly recognized as a potential new therapeutic target in Alzheimer’s disease. In recent years, evidence for Alzheimer’s disease-specific glycoproteins has been established. However, the mechanisms of their dysregulation, including tissue and cell type specificity, are not fully understood. We aimed to explore upstream regulators of aberrant glycosylation by integrating multiple data sources and using a glycogenomics approach. We identified dysregulation by the glycosyltransferase PLOD3 in oligodendrocytes as an upstream regulator in cerebral vessels, and found that it is involved in COL4A5 synthesis, which is strongly correlated with amyloid fiber formation. Furthermore, COL4A5 was suggested to interact with astrocytes via ECM receptors as a ligand. This study suggests directions for new therapeutic strategies for Alzheimer’s disease targeting glycosyltransferases.

## Graphical Abstract

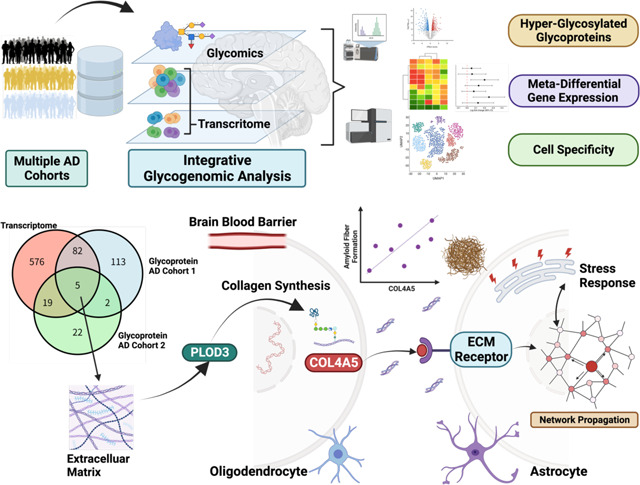

## Introduction

Alzheimer’s disease (AD) is an age-related neurodegenerative disease^1,2^. It’s primary causes are neurogenic cell loss, accumulation of misfolded proteins, oxidative stress, and inflammatory responses^3^. In previous studies, genomic, transcriptomic, epigenetic mechanisms have been intensively examined^4^. However, our knowledge of post-translational modifications that regulate cellular functions and interactions between cells remain still lacking^5^. In particular, glycosylation is the most diverse and abundant post-translational modification among protein modifications^6^.

Glycosylation to proteins is a complex multi-step process involving approximately 200 different glycosyltransferases^6–8^. There are 16 major glycosylation pathways known, including lipid glycosylation, N-glycosylation, O-glycosylation, C-mannosylation, lipid glycosylation, and GPI-anchored synthesis. Recently, glycomics analysis of human AD postmortem brain^9,10^, serum^11–17^ and cerebrospinal fluid^18–20^ for N-glycosylation, the most abundant glycosylation pathway, has revealed a dysregulated glycoproteins. Also, biological functions of abnormal glycans in AD pathology have been reported in some cases, for example, it is known that inhibition of BACE1 glycosylation reduces cleavage of β precursor protein (APP)^21–23^. However, most of the biological functions of glycosylation in the pathogenesis of AD is poorly understood.

Glycan structures themselves are not independent of DNA template, and the glycosylation depends on the combination of about 200 glycosyltransferases and about 500 related proteins^6–8^. Thus, their dysregulation could be an upstream regulatory factor that triggers abnormal glycosylation processes^24^. Besides it is difficult to elucidate biological glycosylation mechanisms at the single cell resolution by glycomics alone since current technology is limited to probing by glycosylation-specific antibodies and glycan-binding proteins (GBP, lectin, etc.)^6^. Therefore a glycogenomics approach that integrates genomics or functional genomics and glycomics is critical for a comprehensive understanding for biological glycosylation pathways^24,25^.

We present upstream factors of aberrant glycosylation in AD. To this end, we performed an integrated analysis of bulk and single-cell/nucleus transcriptomic and glycomics data in human AD brain tissue. In particular, we show that the extracellular matrix (ECM) is a common signature in glycome and transcriptome, and that their expression signatures are enriched for cerebral vascular-related pathways. We identify Procollagen-Lysine,2-Oxoglutarate 5-Dioxygenase 3 (PLOD3) as an upstream glycosyltransferase common to these mechanisms, and through integrated analysis of multiple single-cell expression data. We show that PLOD3 is involved in the regulation of the collagen type IV alpha 5 chain (COL4A5). It is strongly correlated with amyloid fiber formation activity. Cell-cell interaction and signaling pathway analyses suggest that they are involved in the stress response via the ECM receptor on astrocytes.

### Hyperglycosylated proteins are primarily enriched in the extracellular matrix

**Figure 1.**
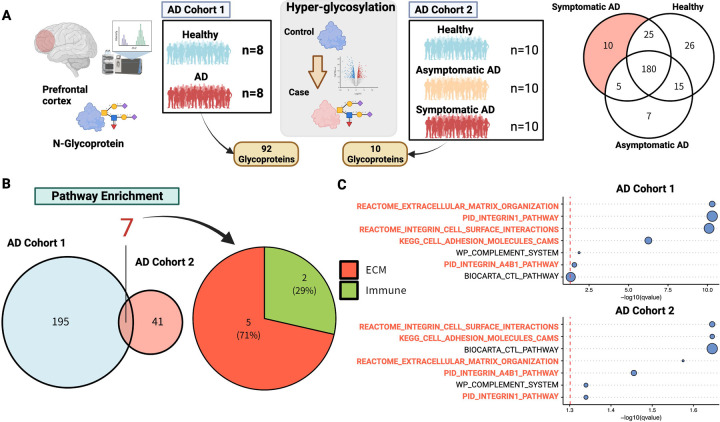
Hyperglycosylated proteins are primarily enriched in the extracellular matrix. (A) Analysis of glycoprotein data from two AD cohorts, using glycoproteins from prefrontal tissues of two independent AD cohorts. The first cohort (AD cohort 1) consisted of 8 samples each from healthy subjects and AD, and the second cohort (AD cohort 2) consisted of 10 samples each from healthy subjects, asymptomatic, and symptomatic AD. In each cohort, 92 and 10 AD-specific glycoproteins were identified. (B) Pathway enrichment of AD-related glycoproteins. Over-representation analysis of AD-specific glycoproteins was performed. (C) Significantly enriched pathways that were common in both cohorts are shown. The horizontal axis is the p-value representing the enrichment, which is the logarithm of the nominal p-value multiplied by a negative value. Figures were created with BioRender.com.

To examine the association between AD molecular pathogenesis and glycosylation, we accessed glycomics data consisting of two cohorts of postmortem brain tissue from AD patients^9,10^ (Figure 1A). The first dataset consisted of eight neuropathologically confirmed AD cases and eight age-matched controls in dorsolateral prefrontal cortex tissue. Another dataset was a subset of the ROSMAP cohort^14^. Glycomics was performed on the postmortem brains of 10 patients with asymptomatic AD, 10 patients with symptomatic AD, and 10 normal brains in which none of the above were present.

In each cohort, 92 and 10 AD-specific hyperglycosylated proteins (Supplementary Table S1) were identified and pathway enrichment analysis was performed on them (Figure 1A). Among the pathways significantly enriched in the two cohorts, we identified the ECM pathway as the most common pathway among 7 pathways (Figure 1B, C). The relationship between Alzheimer’s disease and the ECM has recently been recognized as a new molecular pathogenesis, along with other major pathological hypotheses^26,27^. ECM components contain glycoproteins including glycosylated proteoglycans, collagen as one of major elements^27^, and many glycosylation have important roles in ECM formation and maintenance.

### Meta-analysis of the transcriptome reveals that glycogenes are enriched in the ECM

**Figure 2.**
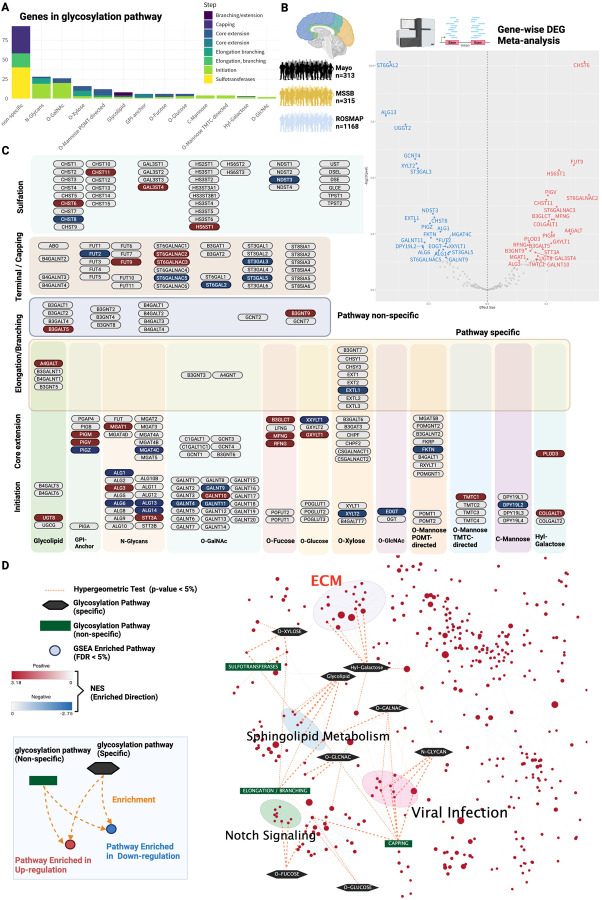
Meta-analysis of the global transcriptome reveals that glycogenes are enriched in the ECM. (A) Number of glycogenes constituting the glycosylation pathway used for transcriptome analysis. (B) Meta-analysis of differential gene expression in multiple AD cohorts. transcriptome data from three AD cohorts: the Mayo cohort (n=313), the MSSM cohort (n=315) and the ROSMAP cohort (n=1168). A meta-analysis of Differential Expressed Gene (DEG) based on gene-level expression levels (FDR < 5%) was performed; 46 glycogenes were identified as DEGs. In the volcano plot, the horizontal axis represents the effect size summarizing the difference in expression between the non-AD and AD groups across cohorts, and the vertical axis represents the log of the p-value from the meta-analysis (bottom is 10) multiplied by a negative value. (C) Mapped glycosyltransferase DEGs. 46 glycogene DEGs were mapped. Genes overexpressed in the meta-analysis are shown in red, genes underexpressed are shown in blue, and genes that did not show significant mutations are shown in gray. Genes are classified into 16 major glycosylation pathways: initiation, core elongation, elongation/branching, capping, and sulfation. Glycosyltransferases with and without pathway specificity are also distinguished. Figure created by BioRender.com.

We explore upstream factors that regulate ECM hyperglycosylation in AD. We accessed the AD Knowledge Portal (https://adknowledgeportal.synapse.org), which contains postmortem brain transcriptome data from multiple cohorts of Alzheimer’s disease patients, and compiled gene expression data. The glycogene set consisting of 214 glycosyltransferases was defined by the gene list in the Glycogene Database (GGDB: https://acgg.asia/ggdb2/)^28^and literature^6,29,30^ (Figure 2A, Supplementary Table 2). This gene set is also categorized by glycosylation pathway and synthesis step (Figure 2A).

We derived the transcriptional signatures of glycogenes based on meta-analysis. We identified 46 differential expressed genes (DEGs) of glycogenes (Figure 2B, Table S3). We mapped glycogenes to glycosylation pathways to determine which pathways are enriched for DEGs (Figure 2C). In all pathways, glycosyltransferases were differentially expressed (Figure 2C), which implicates signals triggering aberrant glycosylation were already observed at the transcriptional level.

**Figure 3.**
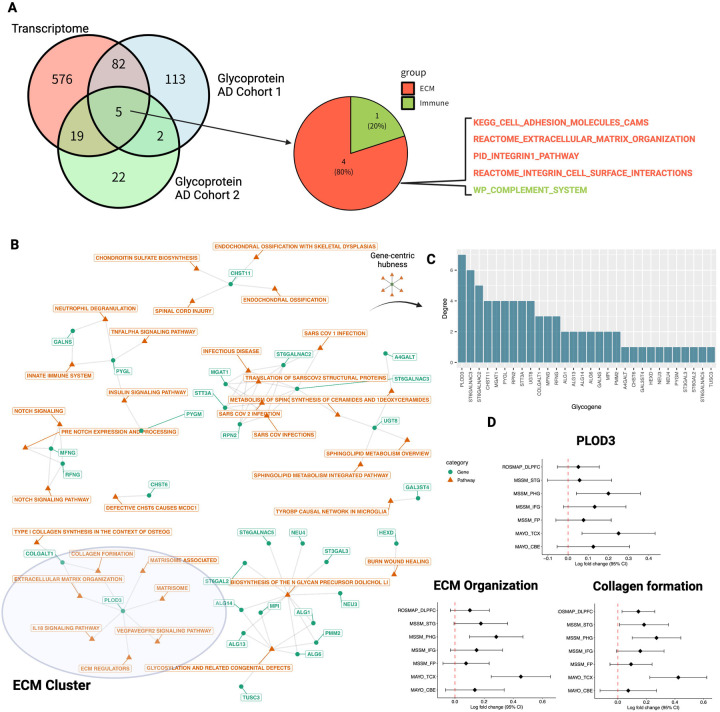
PLOD3 is identified as hub glycogene for ECM. (A) Comparison of AD trascriptome and glycoprotein signatures. Common pathways were shown. (B) Relationship between glycogenes and global enriched pathways. Orange nodes represent globally enriched pathways and green nodes represent glycogen enriched in each pathway. (C) Functional hub glycogenes in globally enriched pathways. To identify functional hub glycogenes involved in multiple pathways, we constructed a pathway-gene bipartite graph, calculated the degree of each glycogene (number of genes directly connected to the pathway), and ranked the importance of each glycogene. The vertical axis of the bar graph represents the order of each glycogene. (D) Activity changes of PLOD3, ECM and collagen formation in AD brains in each transcriptome cohort. Forest plots of log 2-fold changes in PLOD3, ECM organization and collagen formation activity between non-AD and AD are plotted by cohort and brain region. DLPFC stands for dorsolateral prefrontal cortex, STG for superior temporal gyrus, PHG for parahippocampal gyrus, IFG for inferior frontal gyrus, and FP for frontal pole, TCX represents temporal cortex, and CBE represents cerebellum. Dots indicate estimated mean effect sizes, bar widths are 95% confidence intervals of the estimates, and vertical lines with red dots indicate zero (no change).

Next, we analyzed the biological functions for these glycogene signatures. Globally enriched 779 biological pathways were estimated based on the effect size from differential expression obtained by meta-analysis using all genes (FDR < 5%) (Figure 2D, Supplementary Table S2 – S4). Afterward, a post-hoc enrichment analysis was performed to infer which glycosylation pathways are associated with these enriched biological pathways (Figure 2D, Supplementary Table S5). The significant glycosylation pathways with hypergeometric test were extracted as the final estimation results (Figure 2D, Table S4–5; p-value < 5%). We found that the ECM is a common biological signature between layers of transcription and glycosylation in AD (Figure 3A). The ECM cluster was strongly associated with the hydroxyl galactose glycosylation pathway (Figure 2D).

### PLOD3 is identified as functional hub glycogene for ECM

Next, we focused on an in-depth analysis of glycogenes that have a central role for the ECM. Of the 779 globally enriched pathways, we constructed a bipartite graph consisting of glycogene-pathway relationships based on 48 pathways including DEG glycogene (Figure 3B). We inferred glycogene importance via the number of neighboring pathways, i.e., network degree (Figure 3C). As a result, Procollagen-Lysine,2-Oxoglutarate 5-Dioxygenase 3 (PLOD3) was identified as hub glycogene with the highest degree (Figure 3C).

PLOD3 is an enzyme that mediates essential glycosylation in the early stages of collagen formation^31^. In general, collagen is broadly modified by hydroxylation of proline and lysine, and glycosylation of specific hydroxylysine (Hyl) residues^32^. Hydroxylation of lysine is catalyzed by PLOD3^33,34^ and hydroxyllysine undergoes further glycosylation and COLGALT1 transfers galactose, which are critical steps for maintaining collagen integrity^32^.

To further confirm the results at the gene expression levels, we examined whether expression changes of PLOD3 was consistent among the AD cohorts included in the meta-analysis. We found that PLOD3 was consistently upregulated in individual cohort studies (Figure 3D) and similarly, expression signatures of ECM organization and collagen formation also showed a consistently overexpressed trend (Figure 3D). Based on this analysis, we hypothesized that the hyperglycosylation of ECM in AD brain tissue is mediated by PLOD3.

### PLOD3 is expressed in oligodendrocytes and co-expressed with COL4A5

**Figure 4.**
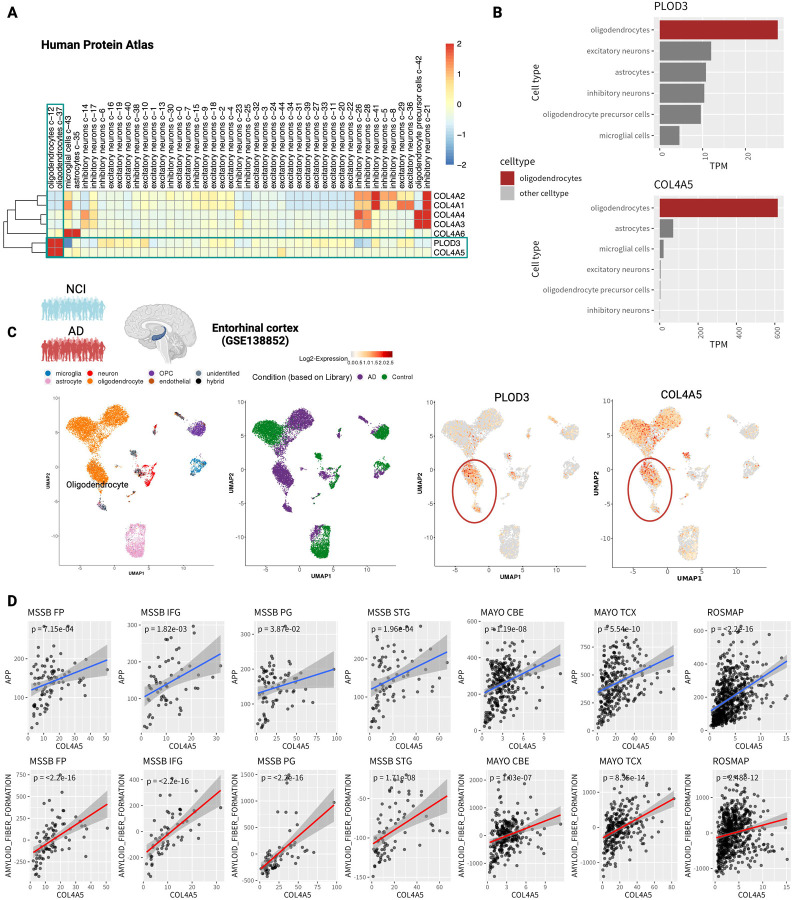
PLOD3 is expressed in oligodendrocytes and co-expressed with COL4A5. (A) Cell type specificity of PLOD3 in healthy brain tissues. Cell clusters obtained from gene expression in healthy brain tissue by Human Protein Atlas (v22) scRNA-seq and the Transcript Per Million (TPM) in each cluster. PLOD3 and COL4A5 are highly expressed in oligodendrocytes and belong to the same cluster. (B) Expression levels of PLOD3 and COL4A5 per cell type. (C) Cellular specificity of PLOD3 and collagen in the enthorhinal cortex. Scatter plots show the cluster structure of cell populations projected by UMAP to 2D coordinates based on gene expression; the first panel shows cell types, the second non-AD and AD; the third and fourth panels show cell type-specific expression of PLOD3 and COL4A5 in oligodendrocytes. (D) Correlation of COL4A5 with expression of APP (upper panel) and activity of amyloid fiber formation (lower panel) for each cohort and each region. DLPFC stands for dorsolateral prefrontal cortex, STG for superior temporal gyrus, PHG for parahippocampal gyrus, IFG for inferior frontal gyrus, and FP for frontal pole, TCX represents temporal cortex, and CBE represents cerebellum.

We sought to determine the cellular origin of PLOD3 and collagen genes. First, we accessed scRNA-seq data of normal brain tissue from the Human Protein Atlas (v22)^35,36^. We found that PLOD3 exhibits a co-expression pattern with collagen type IV alpha 5 chain; COL4A5 in oligodendrocytes (Figure 4A). These two genes showed distinct expression signatures in an oligodendrocyte-specific manner (Figure 4B). We also accessed human AD cohort of single-nucleus RNA-seq (snRNA-seq) data for the entorhinal cortex (GSE138852)^37^. The entorhinal cortex is one of the brain regions that shows neurodegeneration in the early stages of AD^38–40^. The cohort included both non cognitive impairment (NCI) and AD brain. There were six cell types: microglia, astrocytes, neurons, oligodendrocyte progenitor cells (OPCs), oligodendrocytes, and endothelial cells (Figure 4C). PLOD3 and COL4A5 were highly expressed in oligodendrocytes (Figure 4C). They also showed a tendency to be predominantly expressed in the AD group cell population (Figure 4C).

### COL4A5 consistently correlated with amyloid fiber formation in multiple cohort studies

COL4A5 has been partially reported to correlate with amyloid plaque accumulation^41^. However, it has not been validated in large clinical samples. We tested whether COL4A5 significantly correlates with Amyloid Beta Precursor Protein (APP) expression. We again analyzed the bulk RNA-seq data used in the meta-analysis and examined the relationship with APP gene expression, separately for each brain region in each study. The results showed that COL4A5 strongly correlated with the APP gene in all datasets (Figure 4D). Furthermore, we defined gene signatures of amyloid plaque formation pathway and analyzed the correlation between their eigen gene expression and COL4A5 in the same way, and as expected, a strong correlation was confirmed (Figure 4D). PLOD3 was evaluated similarly, showing a weaker correlation than COL4A5, but significant in several data sets (Supplementary Figure 1).

### Cerebrovasculature most strongly associated with ECM dysregulation

**Figure 5.**
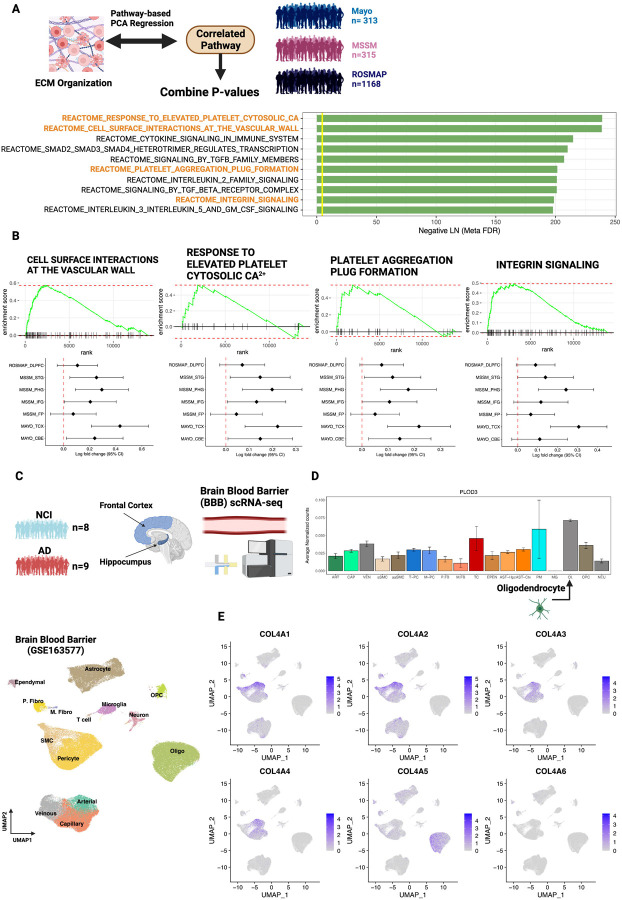
Cerebrovasculature most strongly associated with ECM dysregulation (A) Pathways significantly associated with the activity of the ECM organization were estimated for each cohort tissue using the AES-PCA model. The p-values estimated for each cohort and for each brain tissue were estimated as integrated p-values, and the top 10 pathways are shown in the figure. Figures were generated by BioRender.com. (B) Enrichment of pathways involving the cerebrovasculature in AD with GSEA (FDR < 5%). Forest plots shown below each enrichment plot indicate Log2 fold change for each pathway in each cohort and each region. DLPFC stands for dorsolateral prefrontal cortex, STG for superior temporal gyrus, PHG for parahippocampal gyrus, IFG for inferior frontal gyrus, and FP for frontal pole, TCX represents temporal cortex, and CBE represents cerebellum. (C) Analysis using cerebrovascular scRNA-seq data (8NCI, 9AD). (D) Expression of PLOD3 per cell type (E) Expression of type 4 collagen per cell type.

We explored whether overexpression of the PLOD3 - COL4A5 axis is involved in biological processes in the AD brain. First, we analyzed the biological pathways that best explain ECM activity. We used the AES-PCA^42–44^, a principal component analysis (PCA)-based regression model with ECM activity as the outcome variable and all other biological pathways’ activities as predictors for each AD cohort used in meta-analysis (Figure 5A, Supplementary Table S6). The estimated p-values were statistically combined with fisher’s method (Figure 5A). Four of the top 10 enriched genes were associated with the vascular system (Figure 5A) and they were overexpressed molecules in the AD group at the expression level (Figure 5B). We hypothesized that the PLOD3-COL4A5 axis is involved in the cerebrovascular microenvironment.

### PLOD3 and COL4A5 expressed in oligodendrocytes of the cerebrovasculature microenvironment

We analyzed recently reported scRNA-seq data in the vascular microenvironment in human brain (GSE16357)^45^. This data quantified gene expression by VINE-seq in cerebral blood vessels in 8 NCI and 9 AD (Figure 5C). Gene expression was quantified in 143,793 cells of 14 cell types, including vascular endothelial cells (arterial, capillary, and venous), mural smooth muscle (SMC), pericytes, astrocytes, macrophages, T cells, perivascular and medullary fibroblasts (Figure 5C). We examined cell types expressing PLOD3 and COL4A5, respectively. They were found to be most strongly expressed in oligodendrocytes (Figure 5D and 5E). In contrast, the other type 4 collagens were expressed mainly in pericytes and SMCs, consistent with the fact that type 4 collagen constitutes the vascular basement membrane^46^.

### Oligodendrocytes interact with astrocytes via COL4A5 ligand

**Figure 6.**
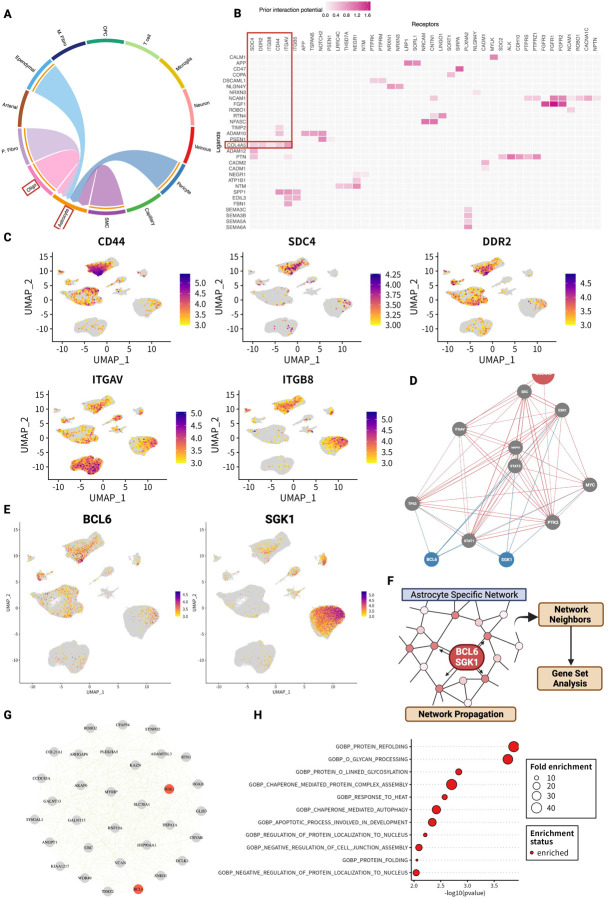
COL4A5 ligand is involved in the regulatory cascade of astrocyte stress response. (A) Estimated cell-to-cell communication based on ECM ligand - receptor expression (B) Receptor candidates for oligodendrocyte-derived COL4A5 ligands predicted to bind in astrocytes. (C) Cell type-specific expression levels of receptors for COL4A5. (D) COL4A5-mediated signaling pathways and target genes in astrocytes. (E) Expression levels of target genes BCL6 and SGK1 per cell type. (F) Analysis flow of the exploration of neighboring genes and functional estimation using network propagation in astrocyte-specific co-expression networks. (G) Top 30 neighboring genes estimated by network propagation based on BCL6 and SGK1. (H) Gene set analysis of BCL6 and SGK1 neighbor genes.

We next analyzed the biological functions and pathways mediated by the PLOD3-COL4A5 axis in cerebrovasculature microenvironment. According to the KEGG pathway, COL4A5 may contribute to cell-to-cell communication via ECM ligand receptors (hsa04512). We analyzed how the PLOD3 - COL4A5 axis of oligodendrocytes mediates intercommunication between cell types. CellChat^47^ allows estimation of cell-cell interactions for each signaling pathway. We estimated cell-cell interactions based on collagen signaling pathways in the AD group. Oligodendrocyte interacted with astrocytes via COL4A5 ligand and CD44 receptor (Figure 6A). It was also checked by NicheNet^48^, another intercellular communication estimation algorithm. In oligodendrocytes, COL4A5 was again identified as one of the most promising candidates (Figure 6B). In addition to CD44 identified by CellChat, SDC4, DDR2, ITGB8, and ITGAV were predicted as receptors in astrocytes (Figure 6B). These receptors were highly expressed in astrocytes (Figure 6C).

### COL4A5 ligand is involved in the regulatory cascade of astrocyte stress response

We further performed a detailed analysis of signaling pathways to understand the biological functions of COL4A5-mediated interactions between oligodendrocytes and astrocytes. We integrated the predicted COL4A5 ligand-receptor pairs (CD44, SDC4, DDR2, ITGB8, ITGAV) onto prior knowledge of the signaling network constructed from multiple perturbation experiments and databases with NicheNet. The results indicated COL4A5 ligand can target and activate B-cell/CLL lymphoma 6 (BCL6) and Serum and glucocorticoid-regulated kinase 1 (SGK1) via the ECM receptors in astrocytes (Figure 6D). BCL6 is a transcription factor and is a known master regulator of humoral immunity and B-cell lymphomagenesis, while SGK 1 encodes a serine/threonine protein kinase and plays an important role in cellular stress responses^49–51^. Both of these genes were also found to be expressed in astrocytes (Figure 6E).

Based on these results, we inferred the biological functions of gene modules with BCL6 and SGK1 in astrocytes. An astrocyte-specific co-expression network was constructed from gene expression using hdWGCNA algorithm^52^ (Figure 6F). Next, we applied the random walk with restart (RWR) algorithm^53^, which is one of the network propagation algorithm, starting from BCL6 and SGK1 on the astrocyte-specific network topology (Figure 6F). The RWR allows the evaluation of the proximity on the network between BCL6 and SGK1 and other neighboring genes. Based on the results, we prioritized top 30 neighbors (Figure 6G). GO analysis on these neighbor gene groups revealed that they are enriched mainly for processes involved in stress response (Figure 6H). These enriched pathways were also observed in the GO analysis of BCL6 and SGK1, which were independently identified using the network propagation method (Supplementary Figure 3).

## Discussion

Our knowledge of how glycosylation, one of the major post-translational modifications, is involved in the pathogenesis of Alzheimer’s disease is lacking. We have systematically explored pathogenesis and driving factors based on integrated analysis of emerging dimensions of glycosylation in combination with transcriptomics.

In brain tissue from human Alzheimer’s disease (AD) patients, hyperglycosylation in the extracellular matrix (ECM) is the main signature shared by glycome and transcriptome, and the glycosyltransferase Procollagen-Lysine,2-Oxoglutarate 5-Dioxygenase 3 (PLOD3) was found to be an upstream regulator acting as a functional hub. PLOD3 was predominantly expressed in oligodendrocytes in AD brain tissue and cerebrovasculature, and was co-expressed with collagen type IV alpha 5 chain (COL4A5). Importantly, COL4A5 significantly correlated with Amyloid Beta Precursor Protein (APP) and the activity of the amyloid fiber formation pathway. Single-cell / nucleus analysis revealed that COL4A5 was a ligand for oligodendrocytes that can mediate cell-cell interactions via ECM receptors on astrocytes. Besides, signaling pathway network analysis identified BCL6 and SGK1 as its target genes and their neighboring genes on the astrocyte-specific network analysis revealed that these two genes are involved in the regulation of stress response.

The involvement of the ECM in Alzheimer’s disease has been supported by a large amount of literature^27,54–58^. The physiological roles of the ECM are diverse and include developmental regulation, tissue homeostasis, cell migration, cell proliferation, cell differentiation, neuronal plasticity, and neurite growth^59^. In AD, in particular, it is known to be extensively involved in perineuronal network (PNN) dysregulation^58,60–68^, which is involved in maintaining spatial structure, neuronal plasticity, scaffolding^69^ and promoting or inhibiting aggregation, as well as in amyloid protein^27,70–75^ and the brain-blood barrier (BBB)^41,54,76–79^. As glycoproteins are one of the major components of the ECM^55,59,80^, the glycan synthesis is clearly important for ECM homeostasis in brain. The enrichment of dysregulated glycoproteins in the ECM is natural in this sense (Figure 1A, B).

We discovered that PLOD3 was enriched for ECM, and it was the up-regulated in AD meta-analysis (Figures 2B–D). PLOD3 is known as a multifunctional enzyme, and in addition to its role as a lysyl hydroxylase, it also functions as a collagen galactosyltransferase and glucosyltransferase activity^34,81–83^. Although no direct evidence of PLOD3 in AD has been reported, it is known to play an essential role in the formation of collagen, a major component of the ECM^84^. For instance, defects in PLOD3 (or Lysyl hydroxylase 3; LH3) have been implicated in causing inherited connective tissue disorders and have been shown to cause cerebral small vessel injury^85,86^, maintenance of the structural integrity of cerebral blood vessels, regulating inflammatory processes^87^. This enzyme is also a promising biomarker in AD, since its expression has been reported to fluctuate in cell-free RNA expression using blood samples from AD patients^88^.

PLOD3 mediates essential glycosylation during early collagen formation^31^. Type 4 collagen is an essential protein in the cerebral vasculature in Alzheimer’s disease and is responsible for network formation in the basement membrane. Indeed, in our analysis of single-cell expression levels in cerebral vessels, many type 4 collagens (COL4A1/COL4A2/COL4A3/COL4A4) were predominantly expressed in pericytes and SMCs (Figure 5E). On the other hand, COL4A5 behaved differently from other type 4 collagens and was expressed dominantly in oligodendrocytes. Oligodendrocytes have been shown to stably bind to cerebral blood vessels by zonation analysis based on single cell / nucleus sequencing analysis^89,90^ and electron microscopy^91^. Interestingly, data from multiple studies supported that COL4A5 strongly correlates with APP and amyloid fiber formation activity (Figure 4D), suggesting a relationship with amyloid plaque accumulation. This may be relevant because overexpression of type 4 collagen generally leads to an increase in cortical basement membrane thickness and has been implicated in the degeneration of cerebral vascular structures^55^. The functional role of type 4 collagen in AD cerebrovasculature needs to be examined in detail by future studies.

We also performed in silico analysis of cell-cell interactions. COL4A5 functioned as a ligand in oligodendrocyte-astrocyte interactions (Figure 6A). Analysis of the signaling pathway network suggested that this cell-cell interaction may contribute primarily to the stress response via SGK1 or BCL6 (Figure 6D–H). SGK1 is known to be transcriptionally upregulated by cellular stress^49–51^. On the other hand, both factors have also been reported to be involved in inflammatory responses in the central nervous system. Recent studies have shown that the inhibition of SGK1 can suppress the NFκB-mediated inflammatory pathway in glial cells^92^. There is also evidence that BCL6 plays a central role in astrocytes and NF-κB in response to inflammatory stimuli and disorders^93^. Indeed, in our analysis of glycoproteins, it was the immune response pathway that was enriched next to the ECM (Figure 1B, C), and also significantly associated with the ECM organization pathway at the transcriptome level were inflammatory cytokines (Figure 5A and Supplementary Figure S2A). Clearly, inflammatory pathways can be characterized as key signatures in the AD brain, but their mechanisms of action on the stress response remain unclear. Further examination of the mechanisms of BCL6- and SGK1-mediated stress responses would be needed.

Several limitations of this study are described. The first is that the AD glycomics analysis is limited to N-type glycans. Therefore, evidence of hyperglycosylation for ECM should be verified by future studies using comprehensive glycomics data. Second, the AD cohort data used in the meta-analysis was limited to data deposited in AD-knowledge portal. In order to establish a higher level of evidence, data from other large cohort studies should be included in the analysis. Third, single-cell sequencing data are collected from several different data sources, so there is no guarantee that the results necessarily reflect the differential expression results of the bulk sequencing used in the meta-analysis. It is expected that this limitation can be overcome in the future as multilayered omics data are collected, but validation including experimental approaches is needed.

Our results suggest that glycosylation is significantly involved in the pathogenesis of AD in several as yet unclarified mechanisms. Our results also indicate that glycogenomics analysis integrating genetic approaches is a promising method to highlight the biological functions of glycans and the molecular pathogenesis of diseases at single-cell resolution. Data on AD glycomics in human subjects are very limited. However, as glycomics analysis technology matures, it is being applied to various disease areas, and a vast amount of glycomics data will be accumulated in the next decade. The glycogenomics approach will play an important role as a bridge between the established AD genetic pathology and the new dimensional omics’ piece, glycomics, in the near future.

## Methods

### Dataset compilation

The Glycomics datasets were compiled and used from supplementary files published in the respective papers^9,10^. For the AMP-AD transcriptome dataset, the data were obtained from The RNAseq Harmonization Study (RNAseq Harmonization) repository (syn21241740). Single-cell transcriptome data in the entorhinal cortex were obtained from GSE138852. Data on the brain vascular were downloaded from GSE163577.

### Glycoproteomics enrichment analysis

The first glycomics data^9^, was analyzed for glycoproteins overexpressed in the AD group (BRAAK ≥ 5) and the normal group (BRAAK ≤ 2), as defined in the original paper, using the canonical pathway collection of MSigDB (c2.cp.v2022.1 Hs.symbols.gmt) were used for enrichment analysis. All genes were analyzed as background using the fedup package in R and the top 30 significantly enriched pathways were identified. The second glycomics data^9^ was analyzed in the same manner. Comparisons were made between the symptomatic group (BRAAK ≥ 5 and CERAD 1 or 2), the asymptomatic group (BRAAK ≥ 3 and CERAD 1 or 2), and the normal group (BRAAK ≤ 2 and CERAD 4) as defined in the original paper, glycoproteins specifically identified in the symptomatic group were extracted. Enrichment analysis was then performed to identify the top 30 significantly enriched pathways.

### Meta-Analysis

Meta-analysis using RNAseq Harmonization of AMP-AD followed the published AD-CONTROL analysis protocol (https://github.com/th1vairam/ampad-DiffExp/tree/df3efa793f379730bae6d4c9e62910fb2c37e525/gene_level_analysis). First, meta-information was used for data from three cohorts (ROSMAP, MSSM, and MAYO) including seven different brain regions to define patients with definitive late-onset Alzheimer’s disease from a clinical and neuropathological perspective, that is, neurofibrillary changes, neuritic amyloid plaques, and cognitive dysfunction The AD control group consisted of patients with AD.

AD controls were defined as patients with few plaques and neurofibrillary changes and no cognitive impairment; in ROSMAP, LOAD cases were those with a BRAAK of 4 or more, a CERAD score of 2 or less, and a cognitive diagnosis of probable AD with no other causes (cogdx=4), LOAD controls are those with a BRAAK of 3 or less, a CERAD score of 3 or more, and a cognitive diagnosis of “no cognitive impairment” (cogdx=1). For MSBB, LOAD cases were defined as those with a CDR score of at least 1, a BRAAK score of at least 4, and a CERAD score of at least 2. LOAD cases were similarly defined as those with a CDR score of 0.5 or less, a BRAAK of 3 or less, and a CERAD of 1 or less as LOAD controls. In Mayo, cases were defined based on neuropathology, with LOAD cases defined as having a BRAAK score of 4 or higher, whereas LOAD controls were defined as having a BRAAK of 3 or lower.

A meta-analysis using a mixed-effects model was then performed on the differences in expression levels in each gene for each of the seven brain regions in each cohort. Effect sizes were estimated by restricted maximum likelihood (REML) based on standard mean difference (SMD) by Hedge. The metacont function from the meta package of the R language was used for the analysis. p-values were corrected for multiple testing by “fdr” using the p.adjust function from the stats package.

### Enrichment Map

Gene Set Enrichment Analysis (GSEA) was performed on all genes included in the meta-analysis. The gene set was c2.cp.v2022.1.Hs.symbols from the MsigDB collection, which was loaded using Enrichment Map in Cytoscape and drawn with default settings. After drawing, we manually classified the pathways into several categories and drew several clusters on the network. The list of glycan-related genes manually defined for each glycosylation pathway was then analyzed by post hoc analysis using the Hyper Geometric Test and the Wilcoxon test, and pathways with FDR ≤ 5% and significant by two tests were extracted. The pathways that were significant by the two tests were extracted.

### Functional hub glycogene identification

Among the pathways enriched based on the same GSEA results as the Enrichment Map, only pathways containing glycogenes were extracted, and from these, a two-part graph of pathway - glycogene was extracted. Based on the obtained two-part graphs, each gene was ranked based on its degree. The glycogene with the largest degree was defined as the functional hub glycogene. The results of querying the extracted PLOD3 to the String database (v11) are shown in Figure 2D. forest plots of PLOD3 was shown with estimated effect sizes and 95% confidence intervals from the meta-analysis. For pathway activity, GSEA was performed using the R fgsea package with gene ranks for effect sizes for each cohort and c2.cp.v2022.1.Hs.symbols for the gene set, and its Normalized Enrichment Score (NES) was used to forest plots were drawn.

### Cell type specificity of PLOD3

For cell type specificity of healthy tissues, information was obtained from the Human Proteome Atalas (V22) website by entering the gene name. For data on entorhinal cortex, information was obtained by entering gene names from (http://adsn.ddnetbio.com/).

### Pathway-based PCA regression and GSEA

Pathway-based PCA is a principal component analysis (PCA)-based method of analyzing pathways and phenotypic associations^43,44,94^. The R Bioconductor PathwayPCA package^42^ was used. Using region-specific gene expression data from each AD cohort (MAYO, MSSM, and ROSMAP), we specified the mean expression levels of the component genes of the ECM pathway as ECM pathway activity for the objective variable and each pathway other than the ECM pathway for the explanatory variables. The gene set used was c2.cp.v2022.1.Hs.symbols from MSigDB. The pathway names containing “ECM,” “Extracellular,” or “Collagen” were defined as ECM pathways. The genes included in the pathways were defined as signatures.

The p-values of the list of pathways significantly associated with ECM were combined by Fisher’s method to calculate an integrated p-value. For the calculation, the logsum function of the R metapackage^95^ was used and the p-values of the individual data sets were entered for each pathway. In addition, we cross-checked whether significantly related pathways were also sufficiently enriched at the expression level. Focusing on the top 10 pathways, we applied GSEA based on the gene set c2.cp.v2022.1.Hs.symbols from MSigDB using the effect sizes of the 3 cohort meta-analysis as gene rank. To further validate that the top 10 pathway activities tended to increase by cohort and region, the means of effect sizes and confidence intervals were calculated for the signature genes and illustrated as forest plots.

### Analysis of brain vascular with scRNA-seq

The count data were preprocessed using the Seurat package in R. The data were preprocessed using the Seurat package in R. That is, normalization, feature selection with vst, scaling, and Dimensional reduction with PCA and UMAP were performed. Cell types were visualized using those already identified in the original paper^45^. Next, for each cell type, variation analysis among AD and cognitively normal groups was performed with Seurat’s FindMarkes function, and enrichment analysis for the identified groups of differentially expressed genes was performed with R fedup package. The c2.cp.v2022.1.Hs.symbols from MSigDB was used as the gene set to determine which cell types were enriched for ECM-related pathways. We selected gene sets with pathway names containing “ECM,” “Extracellular,” “Matrisome,” or “Collagen “ in the pathway name. The enriched p-values were further transformed as −log10(FDR) from the multiple-test-corrected FDR and were considered as differentially expressed activity signals and visualized by heatmap. The expression levels per cell type were obtained by querying (https://twc-stanford.shinyapps.io/human_bbb/) for PLOD3.

### Cell-cell interaction and signaling network analysis

Cell-cell interactions were analyzed using the R package CellChat^47^ (https://github.com/sqjin/CellChat). Oligodendrocytes - astrocytes identified with CellChat were further analyzed using another algorithm, NicheNet^48^ (https://github.com/saeyslab/nichenetr) was used for analysis. For detailed analysis, the ligand-receptor prior information was input by integrating the ligand-receptor pair information used in CellChat with the ligand-receptor pair information used in NicheNet. It was also used for the signal network analysis. A pre-built model was downloaded (https://github.com/saeyslab/nichenetr/blob/master/vignettes/model_construction.md) and the ligand-receptor information identified in the cell-cell interactions and the expression information identified in cell-cell interactions.

### Astrocyte cell type specific network propagation

For astrocyte-specific network construction using cerebrovascular scRNA-seq, Toplogical Over lap Measure (TOM) was estimated using hdWGCNA^52^, and edges were further defined only if they had a TOM above the 90th percentile as a threshold. The network propagation method was then applied using the R package RandomWalkRestartMH^53^. That is, we performed a random walk restart starting from SGK1 and BCL6 on the obtained network topology. The 30 most relevant neighbors were narrowed down and plotted using the R package igraph. The R package fedup (https://github.com/rosscm/fedup) was used for enrichment analysis. To estimate the transcriptional activity of BCL6, curated regulon information was first obtained using R package DoRothEA^96^, and transcription factor target genes were estimated using the Viper^97^ algorithm. R package decoupleR^98^ was used for the analysis.

## Supplementary Material

1
