# Supplementary material for: Single-cell Glycogenomics Deciphers Links Between Altered Transcriptional Regulation and Aberrant Glycosylation in Alzheimer’s Disease
Source: bioRxiv. 2023 Dec 27:2023.12.25.573290. Preprint. [Version 1] doi: 10.1101/2023.12.25.573290 (PMC10793412; doi:10.1101/2023.12.25.573290)
Supplement: Supplementary file 1 [file NIHPP2023.12.25.573290V1-supplement-1.pdf]

bioRxiv preprint doi: https://doi.org/10.1101/2023.12.25.573290; this version posted December 27, 2023. The copyright holder for this preprint (which was not certified by peer review) is the author/funder, who has granted bioRxiv a license to display the preprint in perpetuity. It is made available under aCC-BY 4.0 International license.

Supplementary Figure 1. Correlation of PLOD3 with activity of APP (upper panel) and amyloid fiber formation (lower panel) for each cohort and each region. DLPFC stands for dorsolateral prefrontal cortex, STG for superior temporal gyrus, PHG for parahippocampal gyrus, IFG for inferior frontal gyrus, and FP for frontal pole, TCX represents temporal cortex, and CBE represents cerebellum.

bioRxiv preprint doi: https://doi.org/10.1101/2023.12.25.573290; this version posted December 27, 2023. The copyright holder for this preprint (which was not certified by peer review) is the author/funder, who has granted bioRxiv a license to display the preprint in perpetuity. It is made available under aCC-BY 4.0 International license.

Supplementary Figure 2. ECM activity is strongly associated with inflammatory cytokines. (A) Enrichment of pathways involving the immune system in AD with GSEA (FDR < 5%). Forest plots shown below each enrichment plot indicate Log2 fold change for each pathway in each cohort and each tissue. (B) Pathways significantly associated with ECM activity obtained by applying AES-PCA for each cohort and region. DLPFC stands for dorsolateral prefrontal cortex, STG for superior temporal gyrus, PHG for parahippocampal gyrus, IFG for inferior frontal gyrus, and FP for frontal pole, TCX represents temporal cortex, and CBE represents cerebellum.

bioRxiv preprint doi: https://doi.org/10.1101/2023.12.25.573290; this version posted December 27, 2023. The copyright holder for this preprint (which was not certified by peer review) is the author/funder, who has granted bioRxiv a license to display the preprint in perpetuity. It is made available under aCC-BY 4.0 International license.

Supplementary Figure 3. COL4A5 ligand is involved in the regulatory cascade of astrocyte stress response. (A) Top 30 neighboring genes estimated by network propagation based on BCL6. (B) Gene set analysis of BCL6 neighbor genes. (C) Top 30 neighboring genes estimated by network propagation based on SGK1. (B) Gene set analysis of SGK1 neighbor genes.

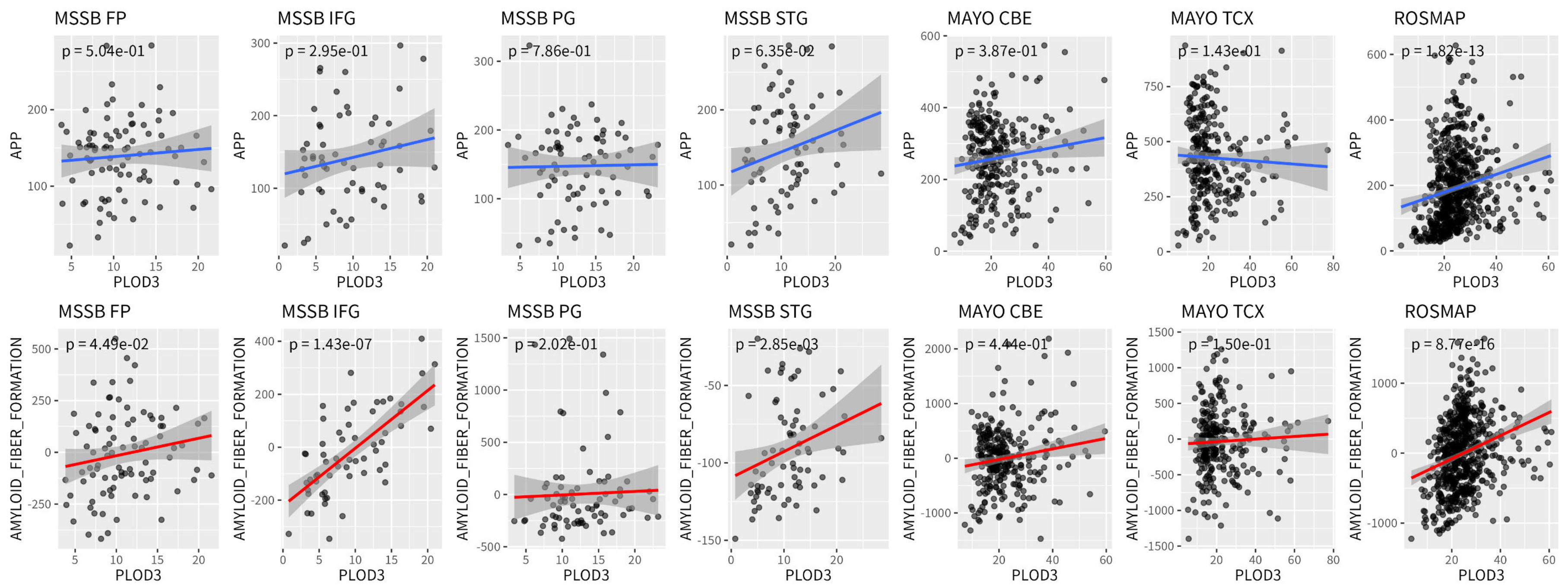

MSSB FP
p = 5.04e-01
MSSB IFG
p = 2.95e-01
MSSB PG
p = 7.86e-01
MSSB STG
p = 6.35e-02
MAYO CBE
p = 3.87e-01
MAYO TCX
p = 1.43e-01
ROSMAP
p = 1.82e-13
APP
PLOD3
MSSB FP
p = 4.49e-02
MSSB IFG
p = 1.43e-07
MSSB PG
p = 2.02e-01
MSSB STG
p = 2.85e-03
MAYO CBE
p = 4.44e-01
MAYO TCX
p = 1.50e-01
ROSMAP
p = 8.77e-16
AMYLOID_FIBER_FORMATION
PLOD3

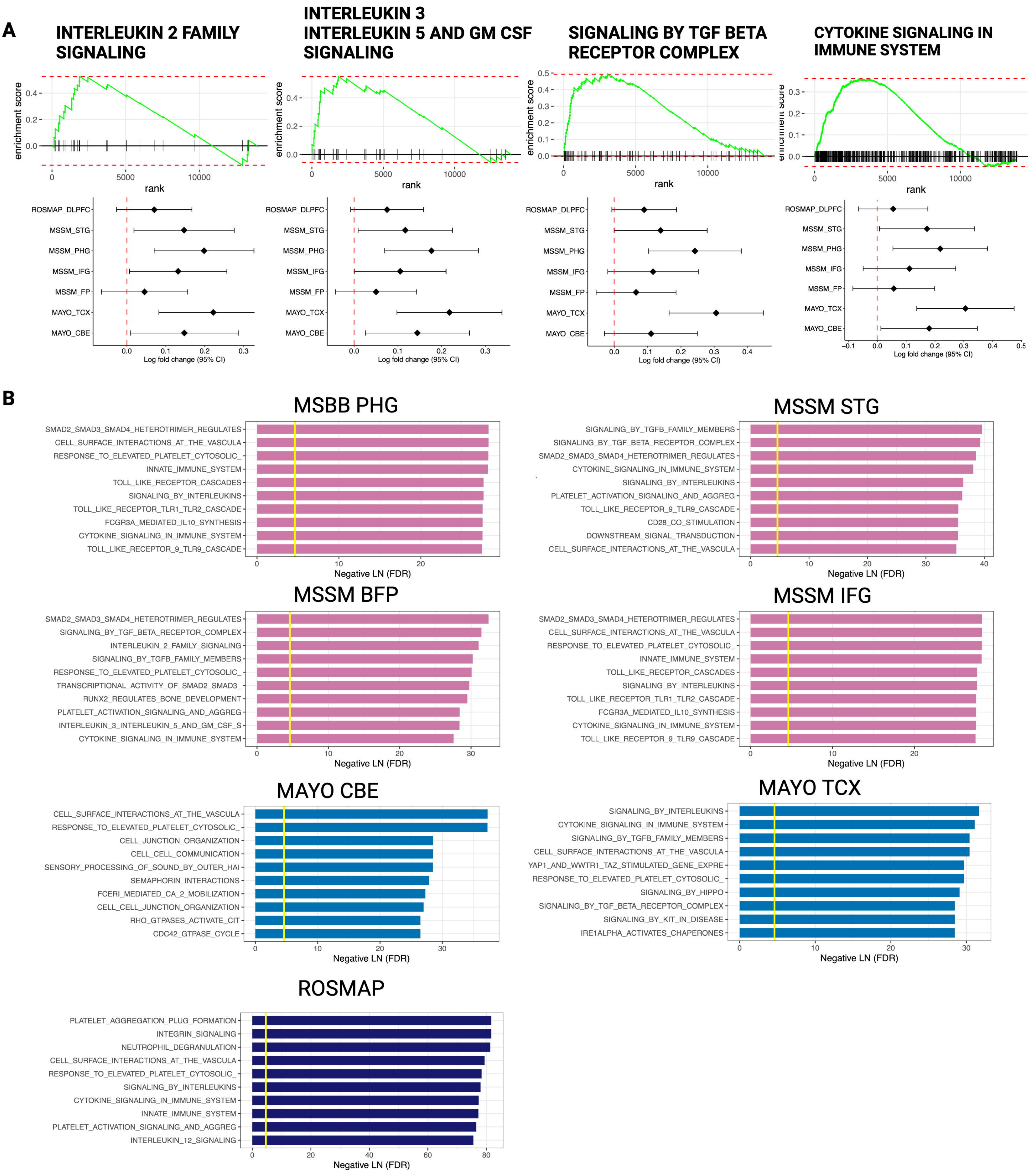
A
INTERLEUKIN 2 FAMILY SIGNALING
INTERLEUKIN 3 INTERLEUKIN 5 AND GM CSF SIGNALING
SIGNALING BY TGF BETA RECEPTOR COMPLEX
CYTOKINE SIGNALING IN IMMUNE SYSTEM
enrichment score
rank
ROSMAP_DLPFC
MSSM_STG
MSSM_PHG
MSSM_IFG
MSSM_FP
MAYO_TCX
MAYO_CBE
Log fold change (95% CI)
B
MSBB PHG
SMAD2_SMAD3_SMAD4_HETEROTRIMER_REGULATES
CELL_SURFACE_INTERACTIONS_AT_THE_VASCULA
RESPONSE_TO_ELEVATED_PLATELET_CYTOSOLIC_
INNATE_IMMUNE_SYSTEM
TOLL_LIKE_RECEPTOR_CASCADES
SIGNALING_BY_INTERLEUKINS
TOLL_LIKE_RECEPTOR_TLR1_TLR2_CASCADE
FCGR3A_MEDIATED_IL10_SYNTHESIS
CYTOKINE_SIGNALING_IN_IMMUNE_SYSTEM
TOLL_LIKE_RECEPTOR_9_TLR9_CASCADE
Negative LN (FDR)
MSSM STG
SIGNALING_BY_TGFB_FAMILY_MEMBERS
SIGNALING_BY_TGF_BETA_RECEPTOR_COMPLEX
SMAD2_SMAD3_SMAD4_HETEROTRIMER_REGULATES
CYTOKINE_SIGNALING_IN_IMMUNE_SYSTEM
SIGNALING_BY_INTERLEUKINS
PLATELET_ACTIVATION_SIGNALING_AND_AGGREG
TOLL_LIKE_RECEPTOR_9_TLR9_CASCADE
CD28_CO_STIMULATION
DOWNSTREAM_SIGNAL_TRANSDUCTION
CELL_SURFACE_INTERACTIONS_AT_THE_VASCULA
Negative LN (FDR)
MSSM BFP
SMAD2_SMAD3_SMAD4_HETEROTRIMER_REGULATES
SIGNALING_BY_TGF_BETA_RECEPTOR_COMPLEX
INTERLEUKIN_2_FAMILY_SIGNALING
SIGNALING_BY_TGFB_FAMILY_MEMBERS
RESPONSE_TO_ELEVATED_PLATELET_CYTOSOLIC_
TRANSCRIPTIONAL_ACTIVITY_OF_SMAD2_SMAD3_
RUNX2_REGULATES_BONE_DEVELOPMENT
PLATELET_ACTIVATION_SIGNALING_AND_AGGREG
INTERLEUKIN_3_INTERLEUKIN_5_AND_GM_CSF_S
CYTOKINE_SIGNALING_IN_IMMUNE_SYSTEM
Negative LN (FDR)
MSSM IFG
SMAD2_SMAD3_SMAD4_HETEROTRIMER_REGULATES
CELL_SURFACE_INTERACTIONS_AT_THE_VASCULA
RESPONSE_TO_ELEVATED_PLATELET_CYTOSOLIC_
INNATE_IMMUNE_SYSTEM
TOLL_LIKE_RECEPTOR_CASCADES
SIGNALING_BY_INTERLEUKINS
TOLL_LIKE_RECEPTOR_TLR1_TLR2_CASCADE
FCGR3A_MEDIATED_IL10_SYNTHESIS
CYTOKINE_SIGNALING_IN_IMMUNE_SYSTEM
TOLL_LIKE_RECEPTOR_9_TLR9_CASCADE
Negative LN (FDR)
MAYO CBE
CELL_SURFACE_INTERACTIONS_AT_THE_VASCULA
RESPONSE_TO_ELEVATED_PLATELET_CYTOSOLIC_
CELL_JUNCTION_ORGANIZATION
CELL_CELL_COMMUNICATION
SENSORY_PROCESSING_OF_SOUND_BY_OUTER_HAI
SEMAPHORIN_INTERACTIONS
FCERI_MEDIATED_CA_2_MOBILIZATION
CELL_CELL_JUNCTION_ORGANIZATION
RHO_GTPASES_ACTIVATE_CIT
CDC42_GTPASE_CYCLE
Negative LN (FDR)
MAYO TCX
SIGNALING_BY_INTERLEUKINS
CYTOKINE_SIGNALING_IN_IMMUNE_SYSTEM
SIGNALING_BY_TGFB_FAMILY_MEMBERS
CELL_SURFACE_INTERACTIONS_AT_THE_VASCULA
YAP1_AND_WWTR1_TAZ_STIMULATED_GENE_EXPRE
RESPONSE_TO_ELEVATED_PLATELET_CYTOSOLIC_
SIGNALING_BY_HIPPO
SIGNALING_BY_TGF_BETA_RECEPTOR_COMPLEX
SIGNALING_BY_KIT_IN_DISEASE
IRE1ALPHA_ACTIVATES_CHAPERONES
Negative LN (FDR)
ROSMAP
PLATELET_AGGREGATION_PLUG_FORMATION
INTEGRIN_SIGNALING
NEUTROPHIL_DEGRANULATION
CELL_SURFACE_INTERACTIONS_AT_THE_VASCULA
RESPONSE_TO_ELEVATED_PLATELET_CYTOSOLIC_
SIGNALING_BY_INTERLEUKINS
CYTOKINE_SIGNALING_IN_IMMUNE_SYSTEM
INNATE_IMMUNE_SYSTEM
PLATELET_ACTIVATION_SIGNALING_AND_AGGREG
INTERLEUKIN_12_SIGNALING
Negative LN (FDR)

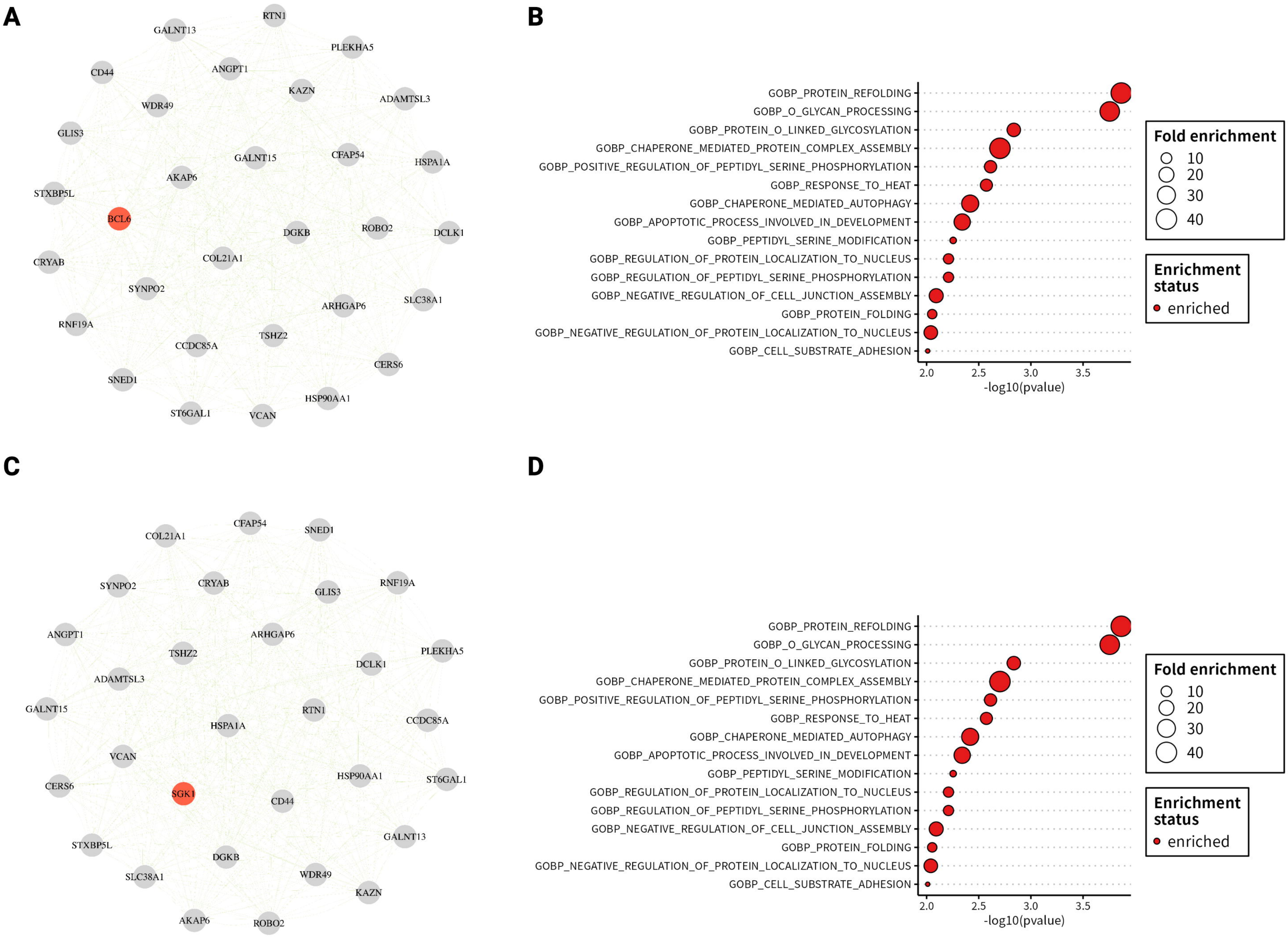

A
RTN1
GALNT13
PLEKHA5
CD44
ANGPT1
KAZN
ADAMTSL3
WDR49
GLIS3
GALNT15
CFAP54
HSPA1A
AKAP6
STXBP5L
BCL6
DGKB
ROBO2
DCLK1
COL21A1
CRYAB
SYNPO2
SLC38A1
ARHGAP6
RNF19A
TSHZ2
CCDC85A
CERS6
SNED1
HSP90AA1
ST6GAL1
VCAN
B
GOBP_PROTEIN_REFOLDING
GOBP_O_GLYCAN_PROCESSING
GOBP_PROTEIN_O_LINKED_GLYCOSYLATION
GOBP_CHAPERONE_MEDIATED_PROTEIN_COMPLEX_ASSEMBLY
GOBP_POSITIVE_REGULATION_OF_PEPTIDYL_SERINE_PHOSPHORYLATION
GOBP_RESPONSE_TO_HEAT
GOBP_CHAPERONE_MEDIATED_AUTOPHAGY
GOBP_APOPTOTIC_PROCESS_INVOLVED_IN_DEVELOPMENT
GOBP_PEPTIDYL_SERINE_MODIFICATION
GOBP_REGULATION_OF_PROTEIN_LOCALIZATION_TO_NUCLEUS
GOBP_REGULATION_OF_PEPTIDYL_SERINE_PHOSPHORYLATION
GOBP_NEGATIVE_REGULATION_OF_CELL_JUNCTION_ASSEMBLY
GOBP_PROTEIN_FOLDING
GOBP_NEGATIVE_REGULATION_OF_PROTEIN_LOCALIZATION_TO_NUCLEUS
GOBP_CELL_SUBSTRATE_ADHESION
2.0
2.5
3.0
3.5
-log10(pvalue)
Fold enrichment
10
20
30
40
Enrichment status
enriched
C
CFAP54
COL21A1
SNED1
SYNPO2
CRYAB
GLIS3
RNF19A
ANGPT1
ARHGAP6
TSHZ2
PLEKHA5
DCLK1
ADAMTSL3
GALNT15
RTN1
HSPA1A
CCDC85A
VCAN
HSP90AA1
ST6GAL1
CERS6
SGK1
CD44
STXBP5L
GALNT13
DGKB
SLC38A1
WDR49
KAZN
AKAP6
ROBO2
D
GOBP_PROTEIN_REFOLDING
GOBP_O_GLYCAN_PROCESSING
GOBP_PROTEIN_O_LINKED_GLYCOSYLATION
GOBP_CHAPERONE_MEDIATED_PROTEIN_COMPLEX_ASSEMBLY
GOBP_POSITIVE_REGULATION_OF_PEPTIDYL_SERINE_PHOSPHORYLATION
GOBP_RESPONSE_TO_HEAT
GOBP_CHAPERONE_MEDIATED_AUTOPHAGY
GOBP_APOPTOTIC_PROCESS_INVOLVED_IN_DEVELOPMENT
GOBP_PEPTIDYL_SERINE_MODIFICATION
GOBP_REGULATION_OF_PROTEIN_LOCALIZATION_TO_NUCLEUS
GOBP_REGULATION_OF_PEPTIDYL_SERINE_PHOSPHORYLATION
GOBP_NEGATIVE_REGULATION_OF_CELL_JUNCTION_ASSEMBLY
GOBP_PROTEIN_FOLDING
GOBP_NEGATIVE_REGULATION_OF_PROTEIN_LOCALIZATION_TO_NUCLEUS
GOBP_CELL_SUBSTRATE_ADHESION
2.0
2.5
3.0
3.5
-log10(pvalue)
Fold enrichment
10
20
30
40
Enrichment status
enriched
